# Combination HIV Prevention among MSM in South Africa: Results from Agent-based Modeling

**DOI:** 10.1371/journal.pone.0112668

**Published:** 2014-11-14

**Authors:** Ron Brookmeyer, David Boren, Stefan D. Baral, Linda- Gail Bekker, Nancy Phaswana-Mafuya, Chris Beyrer, Patrick S. Sullivan

**Affiliations:** 1 Department of Biostatistics, Fielding School of Public Health, University of California Los Angeles, Los Angeles, California, United States of America; 2 Center for Public Health and Human Rights, Johns Hopkins Bloomberg School of Public Health, Baltimore, Maryland, United States of America; 3 Desmond Tutu HIV Centre, University of Cape Town, Cape Town, South Africa; 4 HIV/AIDS, STI/TB Research Programme, Human Sciences Research Council, Port Elizabeth, South Africa, Office of the Deputy Vice Chancellor, Research and Engagement, Nelson Mandela Metropolitan University, Port Elizabeth, South Africa; 5 Department of Epidemiology, Rollins School of Public Health, Emory University, Atlanta, Georgia, United States of America; The University of New South Wales, Australia

## Abstract

HIV prevention trials have demonstrated the effectiveness of a number of behavioral and biomedical interventions. HIV prevention packages are combinations of interventions and offer potential to significantly increase the effectiveness of any single intervention. Estimates of the effectiveness of prevention packages are important for guiding the development of prevention strategies and for characterizing effect sizes before embarking on large scale trials. Unfortunately, most research to date has focused on testing single interventions rather than HIV prevention packages. Here we report the results from agent-based modeling of the effectiveness of HIV prevention packages for men who have sex with men (MSM) in South Africa. We consider packages consisting of four components: antiretroviral therapy for HIV infected persons with CD4 count <350; PrEP for high risk uninfected persons; behavioral interventions to reduce rates of unprotected anal intercourse (UAI); and campaigns to increase HIV testing. We considered 163 HIV prevention packages corresponding to different intensity levels of the four components. We performed 2252 simulation runs of our agent-based model to evaluate those packages. We found that a four component package consisting of a 15% reduction in the rate of UAI, 50% PrEP coverage of high risk uninfected persons, 50% reduction in persons who never test for HIV, and 50% ART coverage over and above persons already receiving ART at baseline, could prevent 33.9% of infections over 5 years (95% confidence interval, 31.5, 36.3). The package components with the largest incremental prevention effects were UAI reduction and PrEP coverage. The impact of increased HIV testing was magnified in the presence of PrEP. We find that HIV prevention packages that include both behavioral and biomedical components can in combination prevent significant numbers of infections with levels of coverage, acceptance and adherence that are potentially achievable among MSM in South Africa.

## Introduction

The identification of a single HIV intervention that is capable of preventing large numbers of infections, such as a highly effective vaccine, remains elusive. Nevertheless, in recent years there have been enormous successes in identifying moderately effective HIV prevention interventions. These interventions include both behavioral and biomedical strategies. The question is how to combine these moderately effective interventions into highly effective prevention packages [1]–[3]. The idea is that multiple interventions when used in combination could prevent more infections than any single intervention used in isolation. Furthermore, the effectiveness of interventions when used in combination may be synergistic.

Although the efficacies of various interventions applied in isolation have been evaluated in a number of rigorous randomized controlled trials, there is little direct evidence about the efficacy or effectiveness of combinations of these interventions [4]–[7]. Estimates of the effectiveness of combinations of interventions are important for guiding the development of prevention strategies. Characterizing the effect sizes of combination prevention interventions are critically important before embarking on large scale randomized controlled trial to rigorously evaluate such combination interventions to insure that the trials are adequately powered [8]–[10].

The drivers of the global HIV MSM epidemics have been previously reviewed highlighting the sustained HIV prevalence and often increasing HIV incidence among these men in several epidemic contexts [11], [12]. The majority of randomized trials in the context of generalized HIV epidemics have focused on heterosexual or vertical transmission of HIV with limited data evaluating efficacy of interventions focused specifically on men who have sex with men (MSM) [13].

Here we report the results from agent-based modeling of the effectiveness of combination prevention interventions for MSM in South Africa. The work is part of the Sibanye Health Project to develop and test HIV prevention interventions among MSM in South Africa. Agent-based modeling has been used to evaluate the drivers of the HIV epidemic in several MSM populations [14]. Here our focus is the use of agent-based models to estimate the overall effectiveness of combination prevention in terms of percentages of infections prevented among MSM in South Africa. Recently, there has been important modeling work of HIV prevention strategies in MSM populations focusing on specific interventions in various regions of the world such as HIV testing in New South Wales [15], circumcision in Peru [16], and antiretroviral treatments including testing and linkage to care (but not pre-exposure prophylaxis) in China [17]. While previous modeling work on combination HIV prevention has been performed in South Africa [18], those models have not focused on the MSM population. This paper is focused on the evaluation of combination HIV prevention in the MSM population in South Africa. We examine four components of combination prevention: treatment of HIV infected persons with ART; prophylactic treatment of high risk HIV uninfected persons to reduce risk of acquisition of HIV infection (PrEP); counseling and condom promotion to reduce the frequency of unprotected anal intercourse; and HIV antibody testing. We perform a detailed statistical analysis of the simulation results of the agent-based model to assess the stochastic variability in the results and to borrow strength across all the simulations to improve estimates of the effects of combination prevention.

## Methods

### Overview of Agent-based Model

Agent-based models are stochastic simulations of interacting agents (e.g. individuals) who may alter behaviors in response to other agents or changes in the environment [19]–[20]. We developed an agent-based model to evaluate combination HIV prevention interventions among MSM in South Africa. Here, we describe the main features of the model which are also summarized in Table 1. Further details and specific model parameter values are given in the Supporting Information S1.

**Table 1:** Main characteristics of agent-based model for combination HIV prevention among MSM in peri-urban South Africa (additional information and specific parameter values are in the Supporting Information S1).

**Attributes assigned to each person at start**
Frequency of sexual activity
HIV status at start
CD4 count at start if HIV +
Knowledge of HIV status at start (yes, no)
Sexual role preference (insertive, receptive, versatile)
HIV testing frequency (3 levels: moderate, low, never)
Some assigned a main partner
Proportion of sexual contacts that are UAI (2 levels)
Sexual networks of regular partners (allowance for sero-sorting)
**Daily updates**
Daily sexual contacts depends on type of partnership
Likelihood of contact (in decreasing order): main, regular, casual, have other main partners
HIV testing possible
UAI rate adjusted if learns knowledge of HIV status
CD4 levels updated for HIV positive
Infection status updated
**Prevention Interventions**
ART for eligible HIV positives
Eligible: HIV test within 6 months and CD4 <350
Considered varying levels of coverage (*X*_1_)
PREP for eligible HIV negatives
Eligible: in last 6 months had both HIV test and >12 UAIs or had infected main partner
Varying levels of PREP acceptance (*X*_2_) with two levels of adherence (low and high)
Reduction in UAI frequency (considered varying reduction levels (*X*_3_))
Increase in HIV testing: convert 50% of the never testers to low frequency testers

Each person (agent) is assigned values for variables associated with risks for transmission and acquisition of HIV. The distributions of these variables were chosen to match available data from South Africa [21]. To simulate the heterogeneities in risk across MSM populations, the values of the variables (e.g., level of sexual activity, numbers of partners) were drawn from probability distributions. The variables include level of and predominant type of sexual activity (e.g., receptive or insertive unprotected anal intercourse [22], numbers of regular partners, whether or not the person is in a main partnership, frequency of HIV antibody test screening, HIV infection status at baseline, and for HIV infected persons, their CD4 levels and whether or not receiving they were receiving ART at baseline. Persons were assigned into variable sized networks of regular sexual partners. One of those regular partners could also be assigned to be the person's main sexual partner. Persons were also allowed to have sexual contact with persons outside their network of regular partners (i.e., casual partners). The probability of sexual contact on any day between two persons depended on whether the partnership was between main partners (most likely), regular partners (somewhat less likely) or casual partners (least likely).

The agent-based simulation proceeded day by day, starting at baseline (which is defined as calendar time *t* = 0). On each day, we simulated whether an HIV uninfected person had sexual contact with an infected person for every possible pair of persons. If an HIV uninfected person had contact with an infected person, we simulated transmission occurrence. The probability of transmission was determined by factors that included the type of sexual contact (e.g., insertive or receptive role in unprotected anal intercourse (UAI)), antiretroviral treatment for the infected partner, and oral Truvada-based pre-exposure prophylaxis (PrEP) for the uninfected partner [5], [23]–[25]. Persons also had an opportunity to receive an HIV test. At the end of each day, the infection status and CD4 cell count were updated [26]. Persons were removed from the simulation when death occurred.

We considered four possible components of combination prevention interventions and different intensity levels of these components. One component was treatment of HIV infected persons with ART [4]. Consistent with current South African national standards, HIV infected persons with a CD4 <350 who had an HIV test within the preceding 6 months were eligible to receive ART. We considered a range of values for the percentage (*X*_1_) of persons eligible for ART and not already in treatment at baseline who receive ART. Here *X*_1_ is measuring additional ART coverage among eligible persons over and above those already receiving ART at baseline. Data from South Africa indicated approximately 50% of eligible persons are on ART and that value was taken to be the baseline level of ART coverage [21].

A second component of combination prevention was prophylactic treatment of high risk HIV uninfected persons to reduce risk of acquisition of HIV infection with tenofovir/emtricitabine (Truvada) (PrEP) [5]. HIV uninfected persons who had an HIV test within the preceding 6 months and were at high risk (defined as either >12 UAI acts in the preceding 6 months or having a main partner who is HIV infected) were eligible to receive PrEP. We considered various values for the percentage (*X*_2_) of eligible persons who were offered and accepted PREP. Persons who received PrEP were classified as either low or high adherers (see online supplement for details). The model allowed adherence level to modify the effectiveness of PREP in reducing risk of HIV acquisition [28].

The third component was counseling and condom promotion to reduce the frequency of UAI [29]. We considered a range of values for the proportionate reduction in UAI contacts (X_3_). For example, *X*_3_ = 15% refers to an intervention that successfully reduces the rate of UAI by 15%. The fourth component was a program to increase HIV antibody testing. We considered an intervention component that decreased by one half the proportion of persons who never receive an HIV antibody test, from 1/3 to 1/6. The presence of this component in combination HIV prevention intervention is indicated by *X*_4_ = 1 (otherwise *X*_4_ is set to 0).

We considered combination prevention interventions consisting of one or more of these four components: ART treatment coverage; PrEP coverage, UAI reduction, and HIV testing increase. We considered a range of for the levels of each component (values for *X*_1_, *X*_2_, and *X*_3_, ranged between 0.0% and 95%, and *X*_4_ took values 0 or 1. For example, a combination HIV prevention intervention with *X*_1_ = 75%, X_2_ = 25%, *X*_3_ = 5%, and *X*_4_ = 1 corresponds to a combination prevention intervention with four components: ART given to 75% of all eligible persons who were not already receiving ART at baseline; PrEP given to 25% of eligible persons; a 5% reduction in the rate of occurrence of UAI; and a halving of the proportion of persons who never received an HIV test. We considered various combination prevention interventions by varying the values of *X*_1_, *X*_2_, *X*_3_ and *X*_4_, and performed multiple replications for each of those combination prevention interventions. We performed replications of the simulations to assess stochastic variation [27].

The number of replications was chosen to control the standard error. Specifically, we calculated the standard error of the mean proportion infected over 5 years after each replication using all replications performed up to that point. If the standard error was above 0.01 we proceeded and performed an additional replication. We stopped replications when the standard error fell below.01. The mean number of replications performed for a combination intervention was 13 with a minimum of at least 5 replications performed for each combination intervention. In addition, we performed 60 replications for the control setting of no intervention (i.e., each *X*_i_ = 0). We considered all packages corresponding to 4 levels each of ART coverage, 4 levels of PREP coverage, 4 levels of UAI reduction and 2 HIV testing levels. In addition we considered a number of additional packages of interest including when one or more of the levels were 0 as well as some additional packages when the UAI reduction was fixed at *X*_3_ = 15% which was a value thought to be potentially achievable. In total we studied 163 HIV prevention packages and a total of 2252 simulations run of the agent-based model across all packages. Each simulation run was carried out for a five year period. The agent-based models were implemented in the statistical programming language **R** with the multithreading package ‘snowfall’ to address the highly intensive computational demands [30]–[31].

### Statistical Analysis

We performed statistical analyses of the dataset of results from the 2252 simulation runs of the agent-based model. The dependent variable (*y*) was the cumulative proportion of MSM that became HIV infected over 5 years from each run of the simulation. We developed a statistical model to relate *y* (the dependent variable) to the levels of the components (*X*_1_, *X*_2_, *X*_3_ and *X*_4_) in the combination prevention intervention (the explanatory variables). We used a generalized linear model with a logistic link of the form, log(*y*/(1-*y*)) which we arrived at after model fitting and regression diagnostics [32]. We considered both linear and higher order polynomial terms (e.g. quadratic and cubic terms) for the levels of the components (the *X*'s). We also considered interaction terms between the components.

Because we performed replications of each combination interventions we were able to evaluate the variance of *y* and found that the variance of *y* was not constant across interventions but varied with the magnitude of *y*. We found that a cubic polynomial adequately described the relationship between the variance of *y* and the expected value of *y*. Accordingly, we used iteratively reweighted least squares to account for the non-constant variance and to estimate the regression coefficients in the model for *y* [33]. We used the resulting model for *y* to calculate the percent of HIV infections prevented for a combination intervention with component levels *X*_1_, *X*_2_, *X*_3_, and *X*_4_ compared to no intervention (i.e., when all the X's are equal to zero). We calculated confidence intervals for the percent of HIV infections prevented that accounted for the covariance between regression coefficients (see Supporting Information S1 for detail). The model allows us to predict the percent of HIV infections that could be prevented among MSM for any levels of the components of combination prevention intervention. The statistical analyses were performed with the **R** programming language [30].

## Results

**Figure 1:**
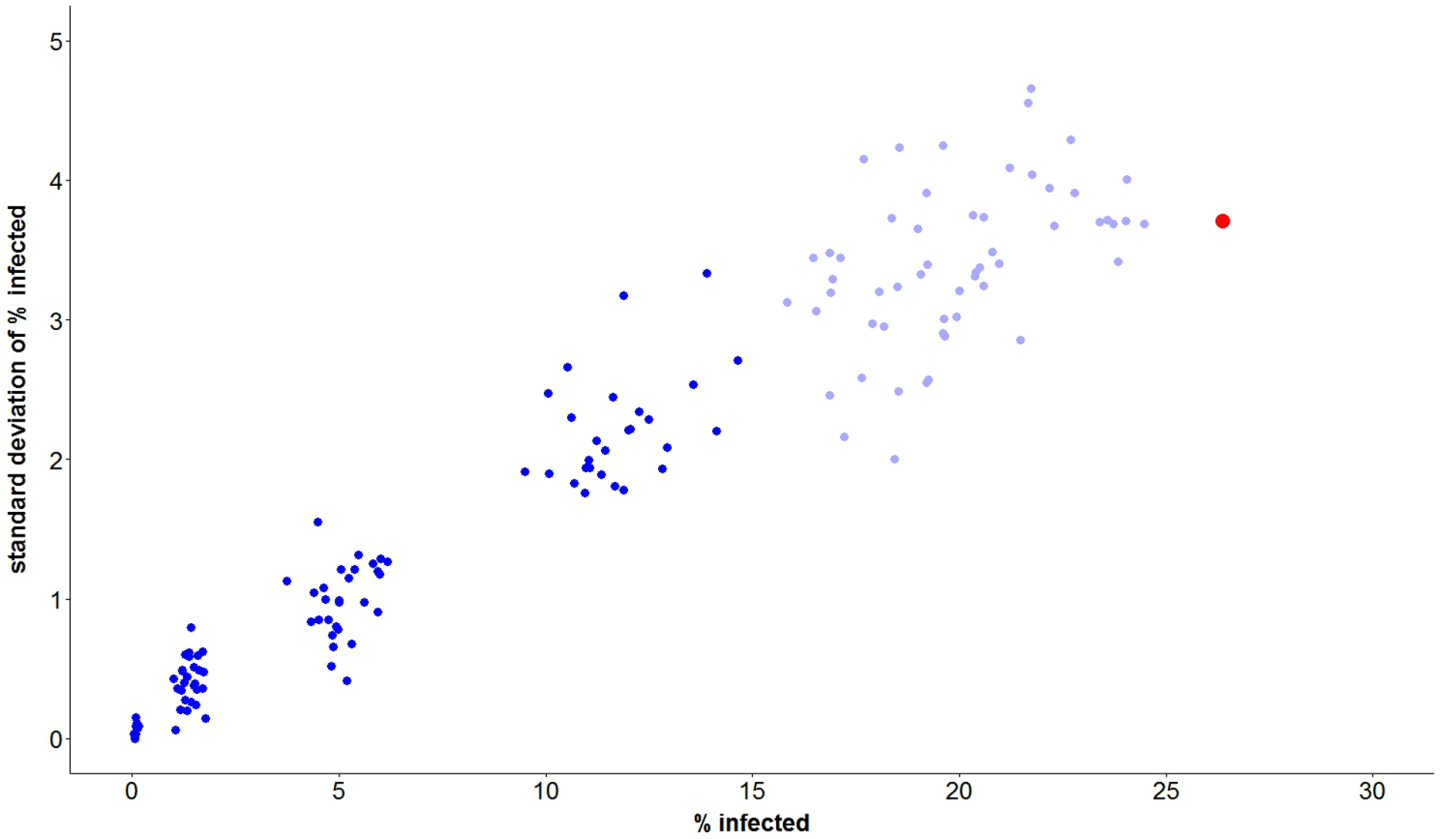
Results from 2252 simulations of agent-based model of HIV spread among MSM in South Africa corresponding to 163 distinct combinations of HIV prevention interventions. Each point represents replicates for a particular combination of HIV prevention interventions. Plotted are the mean percentages infected over 5 years for each intervention (averaged over replicates) versus the standard deviations of those percentages. Combination prevention interventions which included a ≥25% reduction in UAIs are indicated in dark blue, all others are indicated in light blue. The data point in red corresponds to the 60 simulation runs for the control setting of no intervention.

Figure 1 is a graphical display of the 2,252 simulation runs of the agent-based model prior to any statistical modeling of the results. Each data point corresponds to one of the 163 combination prevention interventions including the control setting of no intervention. Each data point plots the average of the cumulative percent of MSM who become HIV infected over five years (*y*) versus the standard deviation of *y* based on all replications performed for that intervention. The mean cumulative percent infected over 5 years ranged as high as 26.4% when there was no intervention (*X*_1_ = *X*_2_ = *X*_3_ = *X*_4_ = 0). As shown in the figure, interventions that reduced the rate of UAI by at least 25% succeeded in reducing the cumulative incidence of infection to less than 15%, and thereby preventing at least 100 x (26.4–15)/26.4 = 43.2% of HIV infections. The figure also shows that the standard deviation of *y* increased with *y*; there was a decreasing trend in the coefficient of variation of *y* (i.e., standard deviation/*y*) from approximately 0.20 to 0.16.

**Figure 2:**
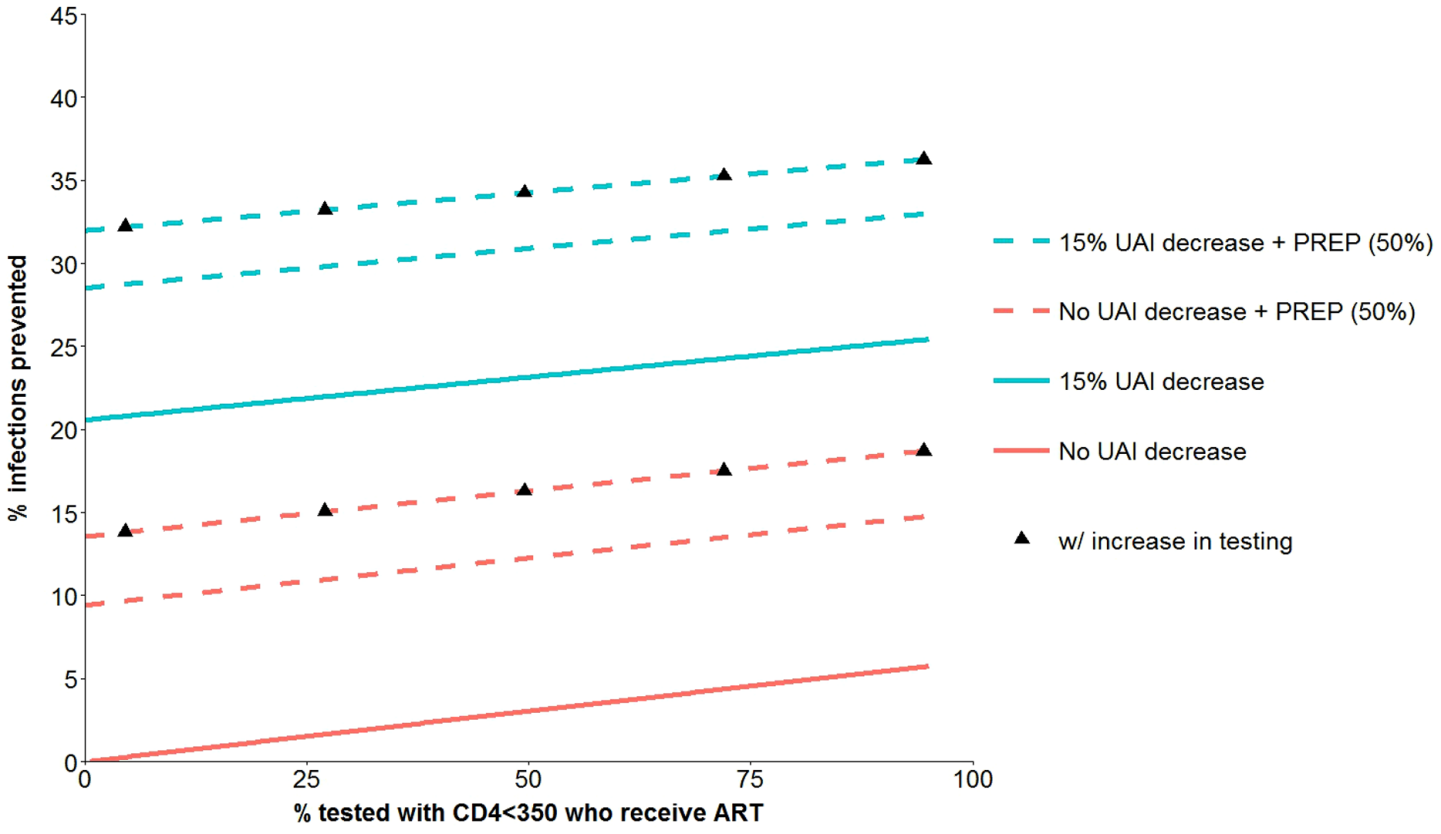
HIV infections prevented over 5 years from combination prevention interventions with four components. ART coverage of eligible persons who were not already receiving ART at baseline, PREP with 50% acceptance (dotted lines), 15% UAI reduction (blue lines; no UAI change are in red) and increase in HIV testing (black triangles). See Table 1 for further details about the components of the prevention interventions.

**Figure 3:**
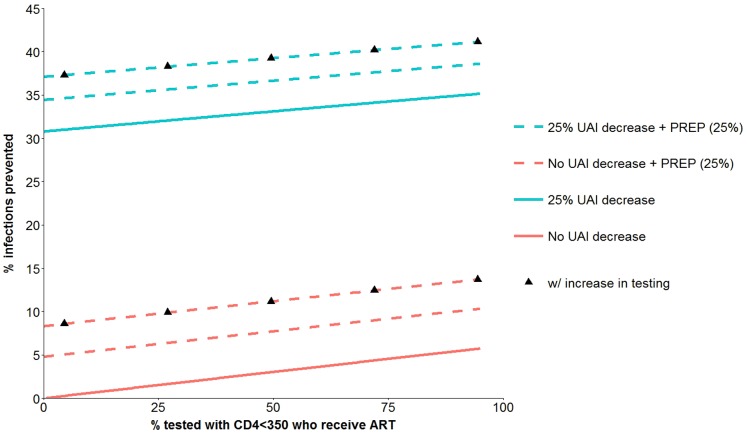
HIV infections prevented over 5 years from combination prevention interventions with four components. ART coverage of eligible persons who were not already receiving ART at baseline, PREP with 25% acceptance (dotted lines), 25% UAI reduction (blue lines; no UAI change are in red) and increase in HIV testing (black triangles). See Table 1 for further details about the components of the prevention interventions.

The regression model equation for *y* is given in the online supplement. Figure 2 is based on that equation and shows the percentage of HIV infections prevented for a range of combination interventions that include: UAI reduction of 0 or 15%; PrEP coverage of 0 or 50%; HIV testing increase that reduced the proportions of persons who never test by 50%; and a continuous range for incremental ART coverage (*X*_1_) over and above persons already receiving ART at baseline. The figure shows that an intervention with only a 15% UAI reduction and no other component was superior to all other interventions that did not include a UAI reduction component. The figure illustrates a positive association between the percentage of infections prevented and increasing ART coverage (*X*_1_), but the positive slope is small and that finding is explained because X_1_, as defined here, refers only to the additional ART coverage of eligible persons (<350 CD4 and an HIV test within the previous 6 months) who were not already receiving ART at baseline. We consider this point further in the Discussion Section. Figure 3 is similar to Figure 2 except it includes interventions with UAI reduction of 25% and PrEP coverage of 25%. The figure shows that a combination prevention intervention with a 25% UAI reduction, 25% PrEP coverage, and HIV testing increase (X_2_ = 25%, X_3_ = 25%, X_4_ = 1) can prevent more than 35% of infections over five years.

To understand how high-impact prevention packages could be constructed, we assumed that a basic prevention package would include ART coverage of 50% of eligible persons. Table 2 shows the effects of sequentially adding components to ART coverage to create HIV prevention packages. We find that the addition of a component that reduces UAI by 15% (*X*_3_ = 15%) would prevent an additional 20.3% of infections over and above the base package (Package 1 in Table 2). That reduction in HIV infections from the addition of a condom promotion/UAI reduction component to the base package is considerably greater than that achieved from the addition of a PrEP component with 50% coverage (which yields an additional 9.5% of infections prevented) or an increase in HIV testing of previously untested men (which yields an additional 2.9% of HIV infection prevented). If we start with a package that includes both 50% ART coverage and 15% UAI reduction components (Package 2 in Table 2), we find that HIV infections could be reduced by 10.1% with the addition of a PrEP component (with 50% coverage) or, alternatively, reduced by 3.1% with the addition of an HIV testing component to reach men never tested for HIV. If we start with a package that includes three components, ART coverage, UAI reduction and PrEP components (Package 3 in Table 2), we find that the addition of the HIV testing component would further reduce infections by 4.9%. We find that the impact of an HIV testing component is greater in the presence of a PrEP component than without a PrEP component (compare 4.9% to 2.9% and 3.1%).

**Table 2:** Incremental contribution from adding components to three prevention packages.

	% infections prevented from adding components		
	to prevention packages (95% CI)^1^		
	Package 1	Package 2	Package 3
	ART	ART & UAI	ART & UAI & PREP
**Additional component**			
UAI (15% reduction)	20.3 (19.4, 21.3)	—	—
PREP (50% of eligible)	9.5 (8.4, 10.6)	10.1 (9.0, 11.2)	—
HIV testing increase	2.9 (0.5, 5.4)	3.1 (0.5, 5.7)	4.9 (1.8, 7.9)

Table presents percent infections prevented from adding a component with 95% confidence intervals (CI). All packages include ART coverage of 50% of eligible persons (from among those not already receiving ART at baseline). Additional components include PREP (50% acceptance of PREP among eligible persons), UAI reduction (15% reduction), and HIV testing increase (50% reduction of persons who have never received an HIV test).

1 The percent infections prevented refers to the percentage decrease in the 5 year cumulative HIV incidence with the HIV package that includes the additional component into the base package.

Table 3 provides information about a four component combination prevention intervention and the contributions of each of the components. The four component package included 50% ART coverage of eligible persons, 50% PrEP coverage of eligible persons, 15% UAI reduction and a 50% reduction in those never tested for HIV. We estimate that this four component package of combination interventions could prevent 33.9% of infections over 5 years (95% confidence interval (31.5, 36.3)) compared to no intervention. We evaluated the incremental impact of each individual component to that four component prevention package by calculating the percent difference in infections with the four component package and comparing that to a three component package that excluded that individual component. For example, the addition of a UAI component to a package that included the other three components (ART, PrEP, and HIV testing) prevented an additional 21% of infections. The UAI component had the largest incremental effect. The addition of the PrEP component to a package consisting of the other three components (ART, UAI reduction, HIV testing) prevented an additional 11.7% of infections. The UAI component had the greatest incremental effect (21%) followed by the PrEP component, while the incremental effects of ART coverage (over baseline levels) and HIV testing were considerably smaller.

**Table 3:** Contribution of four components of an HIV prevention package to infections prevented.

Prevention package component	percent infections prevented due
	to addition of component (95% CI)^1^
ART (50% coverage of eligible persons)	3.4 (2.2, 4.5)
PREP (50% coverage of eligible persons)	11.7 (8.4, 15.0)
UAI (15% reduction)	21.0 (20.0, 22.0)
HIV testing increase	4.9 (1.8, 7.9)
% prevented with all 4 components^2^	33.9 (31.5, 36.3)

Components include ART (50% ART coverage of eligible persons from among those not already receiving ART at baseline); PREP (50% acceptance of PREP among eligible persons); UAI reduction (15% reduction), and HIV testing increase (50% reduction of persons who never have an HIV test).

1 The percent infections prevented due to component *i* refers to the percentage decrease in the 5 year cumulative HIV incidence with the HIV package that includes all four components compared to the HIV prevention package that includes three of the four components leaving out component *i*.

2 The total percent infections prevented refers to the percentage decrease in the 5 year cumulative incidence with the 4 component HIV package compared to no prevention interventions (none of the components). The total percent is not the column sum of the individual components.

## Discussion

In recent years significant progress has been made in HIV prevention science. Findings from HIV prevention trials have demonstrated the effectiveness of behavioral and biomedical interventions such as earlier initiation of ART, PrEP, condoms and behavioral change. The effectiveness of these interventions will depend on their availability in communities as well as levels of uptake and adherence by persons at risk. Each of these interventions is only partially effective in preventing HIV infections, and as such, no single intervention is expected to be sufficient to eliminate the spread of HIV. HIV prevention packages offer the potential to significantly increase the effectiveness of any single intervention. HIV prevention packages offer multiple approaches for reducing risks and possibilities of synergies between the interventions. Quantification of the effectiveness of HIV prevention packages is important for developing combinations of interventions and for designing prevention trials of combination prevention. Unfortunately most research to date has focused on testing only a single intervention rather than HIV prevention packages. In this report we used agent-based models to evaluate the effectiveness of HIV prevention packages among MSM in South Africa.

We identified a four component HIV prevention package for MSM in South Africa which could prevent approximately 34% of infections over five years. We single out this intervention for discussion because it is a potentially achievable combination package that we found to be particularly effective. That four component package consists of 50% ART coverage for eligible persons who were not already receiving ART at baseline, 50% PrEP coverage for high risk eligible persons, 15% UAI reduction and a 50% reduction in those who never test for HIV. The component with the largest incremental impact on infections was the 15% UAI reduction which prevented an additional 21% of infections when added to a package of the other three components. PrEP coverage had the second largest incremental impact, followed by HIV testing and additional ART coverage over baseline levels. We find that even small reductions in UAIs can have huge effects.

We believe the target goals for coverage of each intervention component of the four component package outlined above are achievable in the MSM population in South Africa with concerted commitments and prioritization for HIV prevention for MSM. While the target goals for each component of the prevention package are modest, those goals are at present not being met. Currently, there is essentially no uptake of PrEP among MSM in South Africa; most anal intercourse acts are not protected by condoms; and significant numbers of men in South Africa have not been tested for HIV. In order to achieve the scale of interventions required for public health impact, the coordinated efforts of government, clinicians, and community are required.

We found that the impact of HIV testing in prevention packages depended on which other components were in the package, and specifically, the impact of the HIV testing was magnified when PrEP was included in the package. Such synergies make sense because HIV testing is a gateway to access to PrEP. In this report, we examined the effect of reducing the numbers of persons who never receive an HIV test by half. The effect of an HIV testing component in a prevention package would be greater if the never testers were reduced by more than half or the HIV testing frequencies were increased among persons that do test.

We found a modest effect of ART coverage relative to the other components of the package. This finding was initially surprising because other modeling work has demonstrated that ART can have a significant impact on HIV incidence. For example, Eaton and colleagues [34] performed a systematic review of models to address the question of the impact of ART in a treatment- naïve population. They found that HIV incidence would be considerably lower after 8 years if large numbers remain on ART compared to a counterfactual scenario in which there is no ART. However, Eaton and colleagues emphasize that their result assumed a treatment-naïve population at baseline, that is, the Eaton work assumed “ART was introduced into the population beginning in 2012 with no treatment provision prior to this which is in contrast to the rapid scale up of treatment that has actually occurred prior to 2012 in South Africa” [34]. An important difference of the Eaton work from our work is that we are evaluating the impact of extending ART coverage in the context of significant numbers (50% of eligible persons) already receiving ART at baseline rather than in a treatment-naïve population. Because we had significant numbers of persons receiving ART at baseline, the impact of additional coverage of newly eligible persons is smaller than if the population was treatment- naïve at baseline. ART eligibility requires an HIV test in the preceding 6 months and a CD4 count less than 350. Furthermore, the numbers of persons becoming eligible for ART over the 5 year period (who were not already eligible at baseline) were staggered over the 5 years and were not becoming eligible all at once in a bolus. For example, in our simulation of the control (no intervention) scenario, the number of persons receiving ART at baseline (t = 0) was 55 persons out of 255 infected persons at baseline. In a simulation of the four component package (50% ART coverage for eligible persons who were not already receiving ART at baseline, 50% PrEP coverage for high risk eligible persons, 15% UAI reduction and a 50% reduction in those who never test for HIV), the additional numbers of persons who would go on ART at some time post-baseline during the subsequent 5 years is only 71 persons in addition to the 55 persons already receiving ART at baseline. Furthermore, more than half of these additional 71 persons going on ART would in fact not begin ART until after 2.5 years post baseline (*t*>2.5). These numerical results illustrate that in our simulation work, the incremental ART coverage over baseline is relatively modest. In our modeling setting, the benefit of ART is limited by the number of persons with clinical indication (<350 CD4) who were not receiving ART at baseline and who had an HIV test. As pointed out by Eaton and colleagues “comparing results and conclusions across models is challenging because models have addressed slightly different questions” [34].

The impact of ART is driven by the numbers of treatment at baseline, the treatment threshold, the sufficiency of HIV testing to identify those living with HIV, and of course the extent to which treatment is efficacious in reducing infectiousness. If the guideline for treatment were to shift to CD4 count below 500 or an even higher threshold, the impact of ART would be greater.

The impact of a PrEP component in a prevention package depends on the eligibility requirement. In our work, the PrEP eligibility requirement was either being in a sero-discordant main partnership or having a very high rate of UAIs (12 per 6 months). If that threshold for eligibility is lowered to expand the numbers who are eligible, then the impact of PrEP would be greater than reported here.

We performed considerable numbers of replications of our agent-based modeling to account for stochastic variation. The confidence intervals we report account for the stochastic variation. However, as in all agent-based models, our model is based on numerous assumptions and input parameters. Many aspects of our model such as the networks of sexual partners, distributions of numbers of partners and baseline frequencies of UAIs relied on limited data. Furthermore, the regular and main partners did not change over the 5 years of the simulation. We did however allow persons to have contacts outside their network of regular partners (casual partners). We only forecast five years in an attempt to limit the sensitivity of the results to these model simplifications. More reliable information about partner formation and dissolution among the MSM population on South Africa is important to further inform models of HIV prevention. We focused on the MSM population and did not attempt to model the dynamics of transmission within and across other risk groups such as intravenous drug users. We also did not model variable infectiousness over time. We did not account for new incoming MSM to the population although that simplification may have a small effect over the five years the simulations were run. We recognize that caution should be exercised when interpreting the findings from agent-based models that rely on many simplifications and assumptions. As such, we focused on presenting the results in terms of relative effects of a prevention package (e.g., the percent of infections averted with a prevention package compared to no intervention) because relative effects may be less sensitive to model assumptions than the absolute cumulative number of infections. Nevertheless, caution should still be exercised as with all modeling results. In spite of these limitations, we believe agent-based modeling offers a useful tool for approximating the effectiveness of HIV prevention packages when direct empirical data from comparative studies of combination HIV prevention is unavailable.

Further research on understanding associations within individuals with regard to uptake and adherence levels across the various components of a prevention package will help to refine our models. For example, identification of subgroups that are resistant to accepting or adhering to any intervention would be important for modeling and also for helping to design packages to overcome barriers to acceptance of HIV prevention.

The HIV epidemic among MSM in South Africa continues to grow. Obtaining sufficiently high levels of coverage, acceptance and adherence with any single biomedical or behavioral intervention is a major obstacle to controlling epidemic growth. Combination HIV prevention offers the possibility of preventing significant numbers of infections with sufficient levels of coverage, acceptance and adherence; these levels are achievable with the concerted efforts of multiple stakeholders. In the context of a vigorous debate about the roles of behavioral and biomedical interventions, our results are reconciling in that we demonstrate that traditional HIV prevention activities, such as condom promotion and HIV testing programs, still play vital roles in the context of biomedical prevention. HIV prevention packages that include both behavioral and biomedical components can, in combination, prevent significant numbers of infections among MSM in South Africa.

## Supporting Information

Supporting Information S1 (DOCX)
