# Supplementary material for: Combination HIV Prevention among MSM in South Africa: Results from Agent-based Modeling
Source: PLoS One. 2014 Nov 14;9(11):e112668. doi: 10.1371/journal.pone.0112668 (PMC4232469; doi:10.1371/journal.pone.0112668)
Supplement: Supporting Information S1 — (DOCX) [file pone.0112668.s001.docx]

**SUPPORTING INFORMATION S1**

Each simulated run of the agent based model consists of 1000 persons (agents) whose sexual contacts and infection status are simulated over 5 years. Here we describe in detail the structure of the agent based model, input parameters, and the statistical analysis of the simulation results.

**SPECIFICATIONS OF THE AGENT BASED MODEL**

**Formation of networks and contacts**

We formed networks of regular sexual partners using a logistic model for the probability (*r_ij_*) that persons *i* and *j* are in each other’s network of regular sexual partners. We also used a logistic model for the probability (*c_ij_*) that sexual contact occurs between persons *i* and *j* on a given day. We calibrated the coefficients of the two logistic models above to match South African statistics about partners and contacts as described in more detail below.

The model for *r_ij_* was

$\mathrm{logit}(r_{ij})=-7.18+0.20X_{ij1}+1.099X_{ij2}$ (1)

where *X*_ij1_ is the sum of sexual activity levels for persons *i* and *j*, and *X*_ij2_ indicates whether the infection status of the two partners are the same or not at baseline (calendar time *t*=0). The sexual activity levels for each person are assigned randomly from a probability distribution that matches the number of reported partners in 6 months among South African MSM ( the mean number of unique partners in 6 months is 4.1 and 17.7% of MSM have >5 unique partners in 6 months) (23). Model equation 1 allows for overlapping networks of variable size and a degree of assortive mixing because persons with the same infection status (sero-concordant) are assigned a higher probability of being regular partners than sero-discordant persons. One of a person’s regular partners may be designated as a main partner to match an estimated 46% of MSM in South Africa who are in main partnerships.

The probability that persons *i* and *j* have sexual contact on a given day, *c_ij_*, was determined by

$\mathrm{logit}(c_{ij})=-10.58+10.73M_{ij}+8.847R_{ij}-4.5(H_{i}+H_{j})$ (2)

where the indicator *M_ij_* is set to 1 if persons *i* and *j* are each other’s main partners, the indicator *R_ij_* is set to 1 if persons *i* and *j* are regular (including main) partners, and the indicator *H_i_* is set to 1 if person *i* has a main partner. The model allows for main partners to have higher contact rates than regular partners who in turn have higher contact rates than causal (non-regular) partners.

The regression coefficients of models 1 and 2 were calibrated as follows. We searched over the parameter space of regression coefficients by performing a grid search. For each parameter vector in the gird, we determined how well those parameters produced patterns of sexual activity consistent with known data from the MSM population in South Africa., Specifically, we identified the set of coefficients (for both models 1 and 2 together) that yielded simulation results that best matched the following available statistics on sexual networks and contacts: mean number of unique partners in 6 months=4.1; 17.7% of persons have>5 partners; persons without main partners had 2.7 times more partners than persons with main partners over 12 months. For each parameter vector in the grid search, we ran a simulation for 1.5 years. We calculated simulated values of the above statistics (we used a sliding window over the 1.5 year and performed 10 replications of the simulation . The parameters with the best match in the grid search was determined by minimizing the sum of the squares of the percent difference between the simulated values of the statistics and the statistics on sexual networks and contacts from data from African studies. For example, the simulated value of the number of partners in six months was 3.9 versus 4.1 from the observed data. The regression coefficients were chosen subject to the constraint that the expected number of contacts per week that a person had with main and regular partners were 3.5 and 1.0 respectively. The proportion of contacts that were UAI was adjusted to yield an average annual HIV incidence at baseline of 6% (11-13, 23).

**Inputs and Parameter Values**

Attributes assigned to persons at baseline:

Each person was assigned a set of attributes at baseline (t=0).

- HIV infection status (yes, no); HIV prevalence at *t*=0 is 25.5% (21).
- On ART (yes, no); proportion on ART among HIV infected with CD4<350 at *t*=0 is 0.5 (21).
- Knowledge of infection status (yes, no); proportion with knowledge of HIV status at *t*=0 is 25% (21).
- Sexual role preference: persons are divided equally into three groups, either practicing primarily insertive, receptive, or versatile sexual behaviors with probabilities of receptive behavior on a given contact being 0.2, 0.8 and 0.5 respectively for the three groups.
- HIV testing frequency: Persons are divided equally into three groups corresponding to moderate, low, or never HIV testing frequencies with probabilities of testing within 6 months of .17, .085, and 0.0, respectively. Time between tests follow an exponential distribution. If the CD4 of infected persons drops below 350, persons are moved into the moderate testing group regardless of the initial group assignment.
- Persons are assigned to either low or high propensity groups for engaging in UAI (unprotected anal intercourse) on a given sexual contact with proportions 0.5 in both groups. The probability of engaging in UAI on a given contact for the high and low propensity groups are 1.0 and 0.4, respectively.

Daily Updates:

- Annual decline in CD4 count among HIV infected is 4.26(CD4)^0.5^ where CD4 is current level (26). The CD4 count at baseline ( *t*=0) among HIV positive persons was assumed to follows a lognormal distribution with ln (CD4) ~ N(μ=6.2, σ=0.90). CD4 counts above 1200 are truncated. Persons were assumed to die when the CD4 count dropped below 50.
- If persons learn they’re HIV positive, there’s a 1/3 decrease in their baseline frequency of participating in UAI.
- HIV transmission is simulated by Bernoulli trials using the following transmission rates:

-Transmission probability from infected insertive partner to uninfected receptive partner is 0.014 per contact (23).

-Transmission probability from infected receptive partner to uninfected insertive partner is 0.00175 per contact based on assuming risk of receptive behavior is approximately 8 times that of insertive behavior. (23-25).

-The effect of PREP for uninfected persons is to reduce the probability of transmission by 15% if person is a low adherer to PREP and 73% if person is a high adherer to PREP. Proportions assigned to the high and low adherers groups are each 0.5 (5, 28, 34).

-The effect of ART for HIV uninfected persons is to reduce the probability of

transmission by 96% (4).

Inputs for Network formations:

- Mean number of unique partners in 6 months=4.1; 17.7% of persons have>5 partners (21); persons without main partners had 2.7 times more partners than persons with main partners over 12 months
- Expected number of contacts per week that a person had with main and regular partners were 3.5 and 1.0 respectively. The proportion of contacts that were UAI was adjusted to yield an average annual HIV incidence at baseline of 6% (21).

**Statistical Analysis of Simulation Results from Agent Based Model**

We fit a generalized linear model to the data set of 2157 simulation results. For each of the simulation runs we have the dependent variable *y* which was the proportion infected over 5 years and the explanatory variables being the levels of the components of the intervention package. We used a logit link function.

The modeled percentages infected over five years is given by

$\hat{y}=100e^{Z}/\left(1+e^{Z}\right)$ (3)

where

$Z=\beta_{0}+\beta_{1}X_{1}+\beta_{2}X_{2}+\beta_{3}\tilde{X}_{3}+\beta_{4}\tilde{X}_{3}^{2}+\beta_{5}\tilde{X}_{3}^{3}+\beta_{6}X_{4}+\beta_{7}X_{2}X_{4}$ (4)

and the *X*’s are the covariates that define the components of the combination HIV prevention package. Specifically, *X*_1_ is the percentage of eligible persons who receive ART (from among those not already receiving ART at baseline). Persons are eligible to receive ART if their CD4<350 and had an HIV test within the preceding 6 months; *X*_2_ is the percentage of eligible persons accepting PrEP. Persons are eligible for PrEP if they are HIV uninfected persons, had an HIV test within the preceding 6 months and are at high risk (high risk is defined here as either >12 acts of unprotected anal intercourse (UAI) in the preceding 6 months, or having a main partner who is HIV infected.) $\tilde{X}_{3}$ is a transformation of *X*_3_ where *X*_3_ is the percentage reductions in UAIs (specifically, $\tilde{X}_{3}=\left(100-10\sqrt{100-X_{3}}\right)$; see below for further explanation and motivation for this particular transformation); X_4_ is set to 1 if the percentage of persons who never received an HIV test is reduced in half from 33.3% to 16.7%.

We used iteratively reweighted least squares to account for the non-constant variance of *y*. The estimated regression coefficients in equation 4 were:

**Model component**   **coefficients (β)**

Intercept -1.0906

*X*_1_ -0.000823

*X*_2_ -0.00260

$\tilde{X}_{3}$ -0.04184

$\tilde{X}_{3}^{2}$ 0.000657

$\tilde{X}_{3}^{3}$ -0.00000879

*X*_4_ -0.0391

*X*_2_ *X*_4_ -0.000419

Each of the regression coefficients, β_0_,… β_5_, were individually statistically significant and the main effect and interaction terms for *X*_4_ (β_6_  and β_7_) were jointly significant ( p< .01).

The final variance function that was used for the weighting was

$$var(y)=0.0518\hat{y}-0.1110\hat{y}^{3}$$

where var(y) is the variance of the cumulative proportion infected over 5 years and $\hat{y}$ is the predicted value of *y*.

The confidence intervals for the percent of infections prevented given in Tables 2 and 3 were obtained as follows. Let *ε* represent the proportion of infections prevented with an intervention where the intervention is defined by a row vector of covariates **X**_1_ compared to another intervention that is defined by a different row vector of covariates **X**_2_ As in equation 4, define the linear predictor *Z_1_=****X****_1_* ***β*** and  *Z_2_=****X****_2_* ***β*** where ***β*** is the vector of regression coefficients. Then,

$\varepsilon=\frac{\hat{y}_{2}-y_{1}}{y_{2}}=\frac{\frac{e^{Z_{2}}}{1+e^{Z_{2}}}-\frac{e^{Z_{1}}}{1+e^{Z_{1}}}}{\frac{e^{Z_{2}}}{1+e^{Z_{2}}}}$ (5)

We obtain a confidence interval for *ε* as follows. The first step is to obtain the variance- covariance matrix for the vector of regression coefficients ***β*** from the R programming language which we call *V* (***β***). The second step is to calculate the 2x2 variance-covariance matrix *V*(**Z**) for the two linear predictors *Z*_1_ and *Z*_2_. This covariance matrix is obtained using standard linear statistical theory which gives $V(Z)=\left(\begin{matrix} X_{1} \\ X_{2} \end{matrix}\right)V(\beta)\left(\begin{matrix} X_{1}^{t} & X_{2}^{t} \end{matrix}\right)$ where the superscript *t* represents the transpose. The third step is to calculate the variance of *ε*, called *var (ε),* from the delta method, which gives $\mathrm{var}(\varepsilon)=\left(d\right)V(Z)\left(d^{t}\right)$ where ***d*,** the gradient, is a row vector consisting of the first derivatives of *ε* with respect of *Z*_1_ and *Z*_2_. It can be shown that $d=\left(\begin{matrix} \frac{-\hat{y}_{1}\left(1-\hat{y}_{1}\right)}{\hat{y}_{2}} & \frac{-\hat{y}_{1}\left(1-\hat{y}_{2}\right)}{\hat{y}_{2}} \end{matrix}\right)$. The final step is to calculate a 95% confidence interval for *ε* from

$\varepsilon\pm1.96\sqrt{\mathrm{var}(\varepsilon)}$.

The 95% confidence interval for the percentage of infections prevented is obtained by multiplying the endpoints of the above confidence interval by 100.

The transformation $\tilde{X}_{3}$was used in the model for *y* (equation 4) for two reasons. First, it led to a good fit to the observed dataset of simulation results. Second, it has a mechanistic interpretation. The percent reduction in the probability of the occurrence of UAI on a given contact between two partners from a prevention intervention is *X_3_*. The occurrence of a UAI requires both partners to agree to participate in that behavior. Let $\tilde{X}_{3}$*=* % reduction in an individual’s probability of agreeing to participate in a UAI. Here we develop a relationship between *X_3_* and$\tilde{X}_{3}$. Let *P_0i_* represent the probability that individual *i* agrees to a UAI at *t*=0 (that is, prior to the prevention intervention). Suppose after the prevention intervention that probability is reduced by $\tilde{X}_{3}$ % to become

$\begin{matrix} \text{ }P_{1i}=P_{0i}\left(\frac{100-\tilde{X}_{3}}{100}\right) \\ \end{matrix}$.

Then the probability that a given contact between persons *i* and *j* results in a UAI (assuming independence) is:

$\begin{matrix} \text{ } \\ =P_{0i}P_{0j}\left(\frac{100-\tilde{X}_{3}}{100}\right)^{2} \end{matrix}$.

It follows that the percentage reduction in the probability of occurrence of a UAI (*X_3_*) expressed in terms of$\tilde{X}_{3}$ is

$X_{3}=100-100\left(\frac{100-\tilde{X}_{3}}{100}\right)^{2}$.

Solving the above equation for $\tilde{X}_{3}$ we obtain$\tilde{X}_{3}=\left(100-10\sqrt{100-X_{3}}\right)$.
